# Exploring the Relationship Between Users' Psychological Contracts and Their Knowledge Contribution in Online Health Communities

**DOI:** 10.3389/fpsyg.2021.612030

**Published:** 2021-02-10

**Authors:** Wenlong Liu, Xinting Chen, Xuanyu Lu, Xiucheng Fan

**Affiliations:** ^1^College of Economics and Management, Nanjing University of Aeronautics and Astronautics, Nanjing, China; ^2^School of Management, Fudan University, Shanghai, China

**Keywords:** online health community, transactional psychological contracts, relational psychological contracts, community identification, knowledge sharing self-efficacy, knowledge contribution

## Abstract

The knowledge contribution of members is essential and beneficial to both the business and users of online health communities (OHCs). This study explores and tests the effects of OHC users' psychological contracts on their community identification and knowledge-sharing behavior. A total of 362 valid responses from several well-known OHCs in China are used in the data analysis. The results of the path analysis with structural equation modeling show that users' transactional psychological contracts have a negative effect on their knowledge contribution both directly and indirectly by weakening their community identification. In contrast, users' relational psychological contracts can lead to increased active knowledge contributions both directly and indirectly by enhancing their community identification. Knowledge sharing self-efficacy can strengthen the relationship between relational psychological contracts and knowledge contributions, and the relationship between community identification and knowledge contributions. However, it has no significant impact on the path from transactional psychological contracts to knowledge contribution. The implications and direction of future works are presented on the basis of the results of the empirical analysis.

## Introduction

Knowledge has been recognized as an important strategic resource and a sustainable competitive advantage for individuals and organizations, especially for those in environments with uncertain factors (Wasko and Faraj, 2005; Yu et al., 2010). Knowledge sharing is an activity through which knowledge is exchanged among individuals in an organization to allow recipients to improve their performance (Yu et al., 2010). Today, the rise of the Internet has enabled knowledge sharing and contribution in various ways, such as through online group meetings, which were not possible before, and it also stimulates the development of professional virtual communities that enable participants to share knowledge with one another without ever having to meet in person (Hsu et al., 2007). People are familiar with many of these virtual communities, such as WeChat, weblogs, and specialized virtual communities, including online health communities (OHCs). OHCs are places where people with health problems can obtain a major source of support. These communities allow members not only to retrieve information but also to share their own experiences and communicate with peers that experience the same health problems (Zhao et al., 2014).

Knowledge sharing in OHCs is common today. Over 80% of adult netizens in America use the Internet for health-related purposes. Among them, 34% read the health-related information or comments posted by other users (Zhao et al., 2014). Currently, a significant conduit for knowledge sharing is the conversation exchange among members of these OHCs. In the case of an online community, the knowledge seeker posts an open question or a request for help via a listserv or an online forum; others in the community may either offer an appropriate solution or, if unable to provide an answer, recommend someone else to help (Hara and Foon, 2007). Through this method, the members of these OHCs can acquire many benefits after joining the group. For example, seeking information in OHCs can help users perceive more support, empathy, and optimism from others (Nambisan, 2011). People with serious diseases, such as cancer, can fight their negative feelings because the levels of pressure, depression, and psychological trauma from their health conditions can be reduced after OHC participation (Winzelberg et al., 2003; Beaudoin and Tao, 2008). Moreover, the increase in the demand for health information can lead to the development of tools, which can help users search information more easily and efficiently in return (Nambisan, 2011). Despite increasing measures adopted by online community operators, knowledge contribution continues to be inadequate in OHCs (Ye et al., 2015). Currently, problems regarding knowledge contribution in OHCs hinder facilitators from playing their part.

Knowledge-sharing behaviors in OHCs are affected by community-related factors and personal traits (Anderson, 2004; Hara and Foon, 2007; Ye et al., 2015; Shen et al., 2018). It is found that not only users' perceived community support but also their perceived leader support are positively related to their knowledge contribution (Anderson, 2004; Ye et al., 2015). Some other factors also contribute to facilitating knowledge sharing within the online community, such as self-selection, noncompetitive environment, and the asynchronous nature of the online communication platform (Hara and Foon, 2007). Perceived benefits are believed to directly contribute to individuals' information sharing. Apart from the expectation of users to acquire information from other members, another important aspect of OHC participation is that members pursue spiritual enjoyment, satisfaction, and respect from others. However, perceived risks of privacy, time, energy, and money may lower the level of users' knowledge sharing behavior. Therefore, a secure and user-friendly interaction environment, convenient and fast technology, and effective rewarding mechanisms are significant to enhance the knowledge sharing activities of OHCs (Shen et al., 2018). Moreover, personal outcome expectations also greatly influence knowledge sharing behavior. Once members believe that they can get along better with others by sharing their knowledge, they will be more willing to do so (Bock et al., 2005). Numerous studies have examined the behaviors of knowledge contribution or knowledge sharing in OHCs, in which psychological factors play a very important role. Among them, psychological contracts have been proposed by some researchers. Research on psychological contracts and knowledge sharing by Lan et al. (2020) shows that technical personnel contracts, explicit knowledge sharing, development contracts, and tacit knowledge sharing mutually influence one another—positively or negatively. Affective commitment plays a mediating role in the relationship between the four aspects (Lan et al., 2020).

Psychological contracts involve two major types, namely, transactional and relational contracts (Robinson et al., 1994). Transactional psychological contracts are mainly formed on the basis of monetary or economic aspects with little emotional involvement, thereby leading to low members' identification and spontaneous contribution to the organization (Restubog et al., 2010; Gupta et al., 2012). In contrast, relational psychological contracts, formed based on long-term social cohesiveness, are positively related to members' perceived trust and sense of belonging toward the organization, and in turn result in high willingness to contribute knowledge (Abdullah et al., 2011; Riikka and Läms, 2014). However, most of the research on psychological contracts and knowledge contributions has mainly focused on offline organizations. As a result, few studies are concerned about the significance of psychological contracts on knowledge sharing in virtual organizations. Rousseau (1995) stated that psychological contracts are one's belief, shaped by the organization, with regard to the terms of an exchange arrangement between the individual and their organization. The influences of psychological contracts on members' contributions are of great research value, and their significant role in practical outcomes in the virtual community has also been proposed by scholars (Pirkkalainen et al., 2018; Wei et al., 2018; Bi, 2019). Thus, this study carried out a series of systematic and in-depth investigations in the OHC context.

The objective of our research is to explore the effect of users' psychological contracts on their knowledge contribution in OHCs. The rest of this paper is organized as follows. First, we present the hypotheses and the conceptual model based on the base of our reviewed literature. Second, the measures used in the investigation and some of the data collection procedures are introduced. Then, we illustrate the data analysis techniques and report the results. The theoretical and practical implications of the findings as well as the limitations of this study are disclosed in the final section.

## Literature Review and Hypotheses Development

### Online Health Communities (OHCs) and Psychological Contract Theory

OHC users conduct activities, such as knowledge sharing and member exchanges, on health- or treatment-related issues in the community (Demiris, 2006). By allowing patients to communicate directly with one another, these communities provide an easier and more accessible means of obtaining health information. In recent years, extensive research has been conducted on online health communities. Specifically, users' information search behavior, users' continuous participation behavior, as well as behavior related to user health management in OHCs have drawn a lot of attention (Lin et al., 2015a; Mou et al., 2017; Zhang et al., 2017). Nevertheless, the most common form of activity carried out by participants in online communities is “knowledge sharing” (Hara and Foon, 2007). The information obtained in OHCs can be used by patients to understand their diseases and treatment opinions. Healthy people can also use such information for health risk assessment and disease prevention. Out of consideration of potential threats to online health information, users tend to hide their health-related information. However, the continued growth and prosperity of the OHC depends on whether its members are open and willing to share their personal knowledge (Hui et al., 2007). Therefore, many scholars are concerned about the reasons and incentive mechanisms of knowledge-sharing behaviors in OHCs. It is believed that reciprocity, knowledge self-efficacy, and altruism have positive effects on knowledge sharing intention among health professionals and normal users in OHCs (Zhang et al., 2017). Kuo and Ranganathan (2013) presented a conceptual model of knowledge contribution in OHCs drawing upon three primary factors: individual personality, context, and structural features of the community. In addition to these motivators, self-expression, online reputation, affiliation, sense of belonging, and social capital have been suggested to be key motivators for online knowledge contribution (Chiu et al., 2006; Ma and Agarwal, 2007). The concept of the psychological contract was also used to understand the connection between individual-organization relationships and knowledge contribution motivation (Riikka and Läms, 2014).

A psychological contract refers to an “unwritten contract” that exists between an employee and his/her organization, involving their mutual and implicit expectations from each other (Levinson et al., 1962). Argyris (1960) explored an informal tacit relationship between workers and foremen. His work is the earliest study on psychological contracts. Although he proposed the concept of a psychological work contract, he did not provide its exact definition. On this basis, Rousseau (1990) redefined the early concept of psychological contracts, proposing that the psychological contract is the individual's understanding and belief in the mutual obligations of the employee and the organization; it is also the employee's external and internal contribution. Thus, the psychological contract is an understanding and perception of the exchange relationship between personal contribution and organizational commitments (Rousseau, 1990). MacNeil (Macneil, 1985) first proposed that the psychological contract in an organization includes two components: transactional and relational. Later, Robinson et al. (1994) extracted them as the two dimensions of the psychological contract: transactional dimension and relational dimension. In Riikka's (Riikka and Läms, 2014) research, psychological contracts have been proven useful for finding the best possible method to stimulate the intrinsic motivation of knowledge sharing. Members' psychological contracts are also found strongly related to their participation in the knowledge collaboration in virtual community (Wei et al., 2018; Bi, 2019). However, the impact of psychological contracts on knowledge contribution in OHCs has rarely been investigated despite its important practical significance.

### Psychological Contracts and Knowledge Contribution

Transactional psychological contracts generally emphasize economic, monetary, and materialistic aspects, which comprise factors based on economic exchange (Millward and Hopkins, 1998; Raja et al., 2004; Wei et al., 2018). It describes obligations that are economic and extrinsic, which are more specific and short-term (Gupta et al., 2012). For example, in a company, an employee experiencing a transactional psychological contract is more likely to perceive his/her organization as a source of income and a place to work (Millward and Hopkins, 1998). Researchers have found that transactional psychological contracts, which are highly monetary or materialistic in focus with little close involvement of the parties, do not help facilitate knowledge sharing (Gupta et al., 2012). Moreover, in some enterprises, the situation of knowledge collaboration when most people have transactional psychological contracts is not that active. In the virtual community, the transactional psychological contract dimension refers to a member's self-interest rather than the interest of the whole virtual society (Wei et al., 2018).

Relational psychological contracts mainly depend on trust, loyalty, and job security, which encompass factors based on long-term social cohesiveness (Riikka and Läms, 2014). In addition, it is based on socioemotional exchange and has an open-ended membership involving extensive investments by both parties based on confidence, stability, and high commitment (Taylor et al., 2006). Similar to transactional psychological contracts, relational contracts have special influences on the behavior of people. According to Gupta et al. (2012), relational psychological contracts positively influence knowledge-sharing behavior. The practical implication of this finding is that if an organization expects the engagement of its employees in knowledge sharing, it is vital to facilitate them to build relational psychological contracts with the organization (Gupta et al., 2012). Another study pointed out that relational psychological contracts do not have direct effects on the behavior of knowledge sharing. Specifically, relational psychological contracts are positively related to trust and collaboration, while trust and collaboration are positively related to knowledge sharing. As a result, relational contracts have positive effects on knowledge sharing through trust and collaboration (Abdullah et al., 2011).

In light of these studies, we suppose that these two types of psychological contracts also influence individuals' behavior of knowledge contribution in OHCs and establish the following hypotheses:

Hypothesis 1 (H1). Transactional psychology contracts negatively affect knowledge contribution.

Hypothesis 2 (H2). Relational psychology contracts positively affect knowledge contribution.

### Mediating Effect of Community Identification

Recently, organizations have become increasingly complex and boundless. With this trend, organizational identification is now viewed as an important intrinsic factor for members to increase cohesion and an essential element of organizational success (Smidts et al., 2000; Epitropaki, 2013). Employees who have a strong sense of organizational identification are more inclined to show supportive attitudes toward the organization, and they make their own decisions consistent with the whole organization's objectives (Ashforth and Mael, 1989; Smidts et al., 2000). Companies have recognized that organizational identification has a significant impact on employees' work outcomes, such as job engagement, organizational citizenship behavior, and job performance (Cooper and Thatcher, 2010; Epitropaki, 2013). Moreover, organizational identification is consonant to the belongingness dimension of individuals' perceived organizational membership, that is, to the perception that one has devoted oneself to becoming a member of the organization and a sense of perceived acceptance by the group (Masterson and Stamper, 2003; Epitropaki, 2013).

Rousseau and Tijoriwala (Rousseau and Tijoriwala, 1998) highlighted the dynamic interaction between psychological contracts and organizational identification in their study. Soon afterward, Masterson and Stamper (Masterson and Stamper, 2003) and Stamper et al. (2009) interpreted both constructs within an integrated conceptual framework, which is conceptualized as “perceived organizational membership.” They further suggested perceived organizational membership to be an integrated multidimensional construct reflecting employees' perceptions of their relationship with their organization. Within this framework, psychological contracts have been verified to facilitate employees' perceptions of organizational membership through their perceptions of need fulfillment, whereas organizational identification is consonant to the belongingness dimension of perceived organizational membership (Epitropaki, 2013).

Moreover, psychological contract fulfillment is a crucial predictor of employee satisfaction and organizational identification; specifically, perceptions of psychological contract fulfillment are positively related to organizational identification and job satisfaction. In contrast, psychological contract breach has a negative impact on these outcomes (Rodwell et al., 1971). Similarly, studies also revealed that psychological contract fulfillment has a positive impact on perceived organizational support and thereby may be linked to organizational identification and organizational citizenship behaviors (OCBs) (Zagenczyk et al., 2011; Ahmad and Zafar, 2018). However, in regard to the specific dimension, transactional psychological contracts have a negative effect on both in-role and extra-role performance, which means decreased work engagement, reduced job satisfaction and job commitment, low identification, and high intention to quit (Lu et al., 2016). On the other hand, they also found that relational psychological contracts contribute to higher organizational identification as well as OCBs. Similar results were reflected in the study of Tufan and Wendt (Tufan and Wendt, 2020). They employed a perspective of psychological contract breach and determined that relational psychological contract breach reduces ethnic minority employees' organizational citizenship behavior by damaging their employment relationships and causes these employees to dissociate their identity from that of the organization (Tufan and Wendt, 2020).

Given that all these studies are related to offline organizations, online organizations and communities have rarely been investigated. Drawing on the above literature, we suppose that the two types of psychological contracts have significant influences on community identification in OHCs and set up the following hypotheses:

Hypothesis 3 (H3). Transactional psychology contracts negatively affect community identification.

Hypothesis 4 (H4). Relational psychology contracts positively affect community identification.

Community members actively participate in community activities when they identify with the community (Algesheimer et al., 2005). Tidwell (2005) demonstrated that people increase their contributions if they strongly identify with an organization. Wasko and Faraj (2005) explained that people feel obligated to help others because they have a common membership when they identify with a group. Group identification positively influences knowledge-sharing behavior in the community. Moreover, numerous studies have indicated that if members stay in contact with the community psychologically, they are more inclined to behave in terms of their community sake (Bergami and Bagozzi, 2000; Gruen et al., 2000; Mcwilliam, 2000). In a virtual community, when members accept its culture and values and identify with the community, they acquire a sense of belonging and trust and are more willing to share knowledge in the community. For example, members of a travel community with high identification are more willing to participate in community events to exchange information, opinions, and experiences with others (Ardichvili et al., 2003; Koh and Kim, 2004). van den Hooff et al. (2003) supported this claim by stating that in knowledge communities, members are more likely to share knowledge when they identify strongly with the group. Other studies have also verified that community identification is positively related to knowledge-sharing behavior in the virtual community (Nahapiet and Ghoshal, 1998; Chiu et al., 2015). Moreover, some research has discussed the mediating role of identification. Qu and Lee (2011) demonstrated that participating in the community can enhance users' community identification and promote knowledge sharing via community identification. Restubog et al. (2010) determined that organizational identification plays a mediating role in the relationship between psychological contract breach and organizational citizenship behavior. For OCBs, Ramasamy and Thamaraiselvan (2011) proposed a conceptual framework in which the five components of OCBs are closely linked to spontaneous behaviors and are positively related to knowledge sharing. On the basis of previous studies, we speculate that some underlying mechanisms exist between psychological contracts and knowledge contribution via community identification. This study sets up the following hypotheses:

Hypothesis 5 (H5). Community identification has a positive effect on knowledge contribution.

Hypothesis 6a (H6a). Community identification plays a mediating role in the relationship between transactional psychology contracts and knowledge contribution.

Hypothesis 6b (H6b). Community identification plays a mediating role in the relationship between relational psychology contracts and knowledge contribution.

### Moderating Effect of Knowledge Sharing Self-Efficacy

Knowledge sharing self-efficacy refers to knowledge contributors' confidence regarding whether they have enough ability to provide valuable knowledge to other members (Ye et al., 2015). When community members have high knowledge sharing self-efficacy, they believe they can provide useful information and valuable knowledge. Thus, the users in this community can be more willing to contribute knowledge to the community. Studies have revealed that knowledge sharing self-efficacy has a positive effect on knowledge contribution (Liou et al., 2016; Yilmaz, 2016; Zhang et al., 2017). For example, Hsu et al. (2007) pointed out that individuals' knowledge sharing self-efficacy is positively related to their knowledge-sharing behavior. A low level of perceived knowledge sharing self-efficacy can be a significant obstacle to knowledge contribution behavior because people may doubt whether they have sufficient ability to share information (Hsu et al., 2007). Within the context of OHCs, knowledge self-efficacy is believed to have a more significant influence on the knowledge contributing intention of health professionals than of patient users (Zhang et al., 2017). With the popularity of the Internet, many normal users can use different sources through website links to obtain information and share health knowledge with others (Zhang and Sun, 2015). According to Alessandri et al. (2009), compared to those with low self-efficacy, individuals with a high level of self-efficacy are more inclined to provide support to others and even not hesitate to make certain sacrifices to enact such prosocial behaviors. Rather than the return from their contribution, they fulfill their needs for esteem, self-actualization and even subjective well-being by their contributing behavior (Chiu et al., 2015). Based on the above literature, this study suggests that knowledge sharing self-efficacy can weaken the negative influence of transactional psychological contracts on individuals' knowledge contribution. That is, knowledge sharing self-efficacy negatively moderates the relationship between transactional psychological contracts and knowledge contribution. On the other hand, community members who perceive an emotional connection and a sense of belonging to the community and other members are more inclined to contribute for the sake of the community, and to help other members (Utz and Sassenberg, 2002). Moreover, if they believe themselves to have the capability to help others, they will be more active in contributing their knowledge. Given this, we believe that knowledge sharing self-efficacy positively moderates the relationship between relational psychological contracts and knowledge contribution. Meanwhile, individuals who feel capable of coping with challenges will show a strong intention to offer support to others for the sake of community identification. That is, community identification will have a stronger effect on knowledge contribution for individuals with high self-efficacy than for those with low self-efficacy (Lin et al., 2015b). Therefore, this study suggests that knowledge sharing self-efficacy positively moderates the relationship between community identification and knowledge contribution. To sum up, we propose the following hypotheses:

Hypothesis 7a (H7a). Knowledge sharing self-efficacy negatively moderates the influence of transactional psychological contracts on knowledge contribution.

Hypothesis 7b (H7b). Knowledge sharing self-efficacy positively moderates the influence of relational psychological contracts on knowledge contribution.

Hypothesis 7c (H7c). Knowledge sharing self-efficacy positively moderates the influence of community identification on knowledge contribution.

## Research Design and Methodology

### Measurement

**Figure 1:**
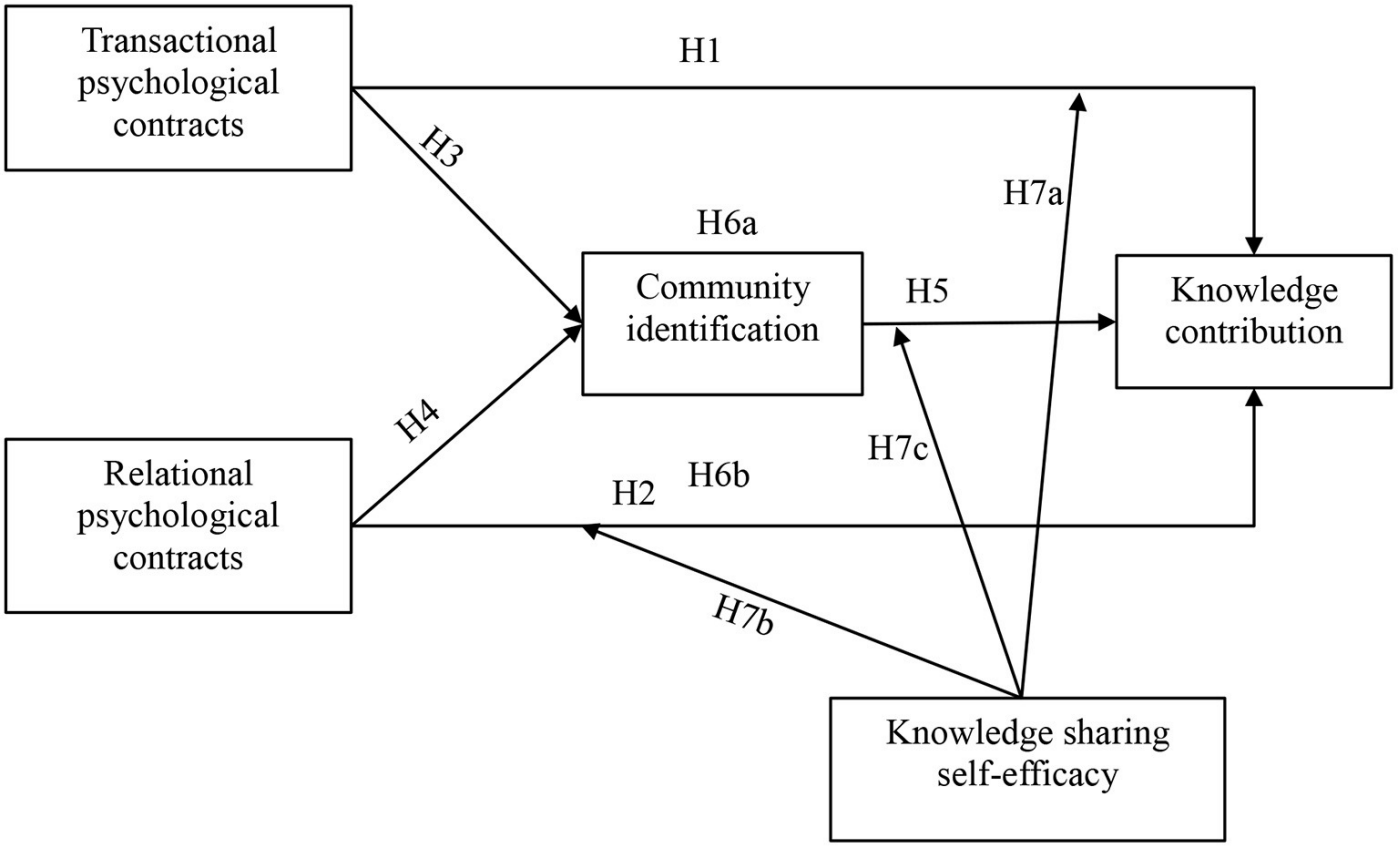
Conceptual model.

A questionnaire is developed for data collection, which consists of scales to measure all the constructs of the research model (Figure 1) and demographic characteristics. All the items for measuring the constructs were adopted from the existing literature and adjusted to fit our OHC context.

We adapted items for psychology contracts from the studies of Soares and Mosquera (2019) and Lu et al. (2016) and modified them on the basis of the concept of psychological contracts in the virtual community context by Wei et al. (2018). The measures of community identification were adapted from Wang and Wei (2011). A sample question is “I feel a sense of belonging to my virtual community.” In addition, we adapted items for knowledge sharing self-efficacy from Kankanhalli et al. (2005). Knowledge contribution was measured by three questions adopted from Ye et al. (2015). A sample question is “I have confidence in responding or adding comments to messages or articles posted by other members in the online health community.” Following the approaches used by Soares and Mosquera (2019), the responses to the questions about transactional psychology contracts were rated on a five-point Likert scale ranging from 1 (strongly disagree) to 5 (strongly agree), and relational psychology contracts were measured on a seven-point Likert scale ranging from 1 (strongly disagree) to 7 (strongly agree). In accordance with Wang and Wei (2011), the responses to the questions about community identification were rated on a five-point Likert scale. Following the approaches used by Kankanhalli et al. (2005) and Ye et al. (2015), knowledge sharing self-efficacy and knowledge contribution were measured on a seven-point Likert scale. Table 1 shows the measures for all constructs.

**Table 1:** Research constructs and measurements.

**Construct**	**Items**	**Source**
Transactional Psychological Contracts (TPC)	TPC1 I am under no obligation to remain with this online health community. TPC2 I participate in community interaction to obtain the information I need. TPC3 I only perform specific duties I had agreed to when I joined TPC4 I spend more energy on answering questions with rewards.	Wei et al., 2018; Soares and Mosquera, 2019
Relational Psychological Contracts (RPC)	RPC1 I feel part of a team in this online health community. RPC2 I expect to grow in this online health community. RPC3 I commit myself personally to this online health community. RPC4 I feel that this online health community reciprocates the effort put in by its members.	Lu et al., 2016; Soares and Mosquera, 2019
Community Identification (CI)	CI1 I feel a sense of belonging to my virtual community. CI2 I am glad that I joined my virtual community for knowledge contribution purposes. CI3 I have a strong positive feeling toward my virtual community. CI4 I am proud to be a member of my virtual community.	Wang and Wei, 2011
Knowledge Sharing Self-efficacy (KSS)	KSS1 I have confidence in my ability to provide knowledge that other members in the online health community consider valuable. KSS2 I have the expertise, experiences, and insights needed to provide knowledge that is valuable for other members in the online health community. KSS3 I have confidence in responding or adding comments to messages or articles posted by other members in the online health community.	Kankanhalli et al., 2005
Knowledge Contribution (KC)	KC1 I help other people in this online health community who need help/information from other members. KC2 I take an active part in helping others in this online health community KC3 I contribute knowledge to this online health community KC4 I contribute knowledge to other members that may result in their development of new insights	Ye et al., 2015

### Survey Administration

Research data were collected among the users of several main Chinese OHCs, such as Dingxiangyuan (http://www.dxy.cn/bbs/index.html) and Manyoubang (http://www.manyoubang.com), from June to August 2020. A total of 491 registered users of these websites participated in the survey. With the exclusion of 129 invalid questionnaires, a total of 362 complete and valid questionnaires were used for data analysis. Table 2 shows the demographic statistics of the respondents.

**Table 2:** Result of demographic statistics analysis (*N* = 362).

**Demographic Profile**	**Category**	**Frequency**	**Percent (%)**
Gender	Male	156	43.1
	Female	206	56.9
Age	Less than 19	21	5.8
	20–29	134	37.0
	30–39	150	41.4
	40–49	44	12.2
	50–59	11	3.0
	60 or above	2	0.6
Education	Education High school or below College	32	8.8
	University	293	80.9
	Graduate school or above	37	10.2
Duration	Less than 3 months	91	25.1
	3−6 months	90	24.9
	6 months to1 year	91	25.1
	1−2 years	52	14.4
	2 years or above	38	10.5
Frequency	Less than once a month	27	7.5
	Once a month	90	24.9
	A few times a month	96	26.5
	A few times a week	69	19.1
	About once a day	47	13.0
	Several times a day	33	9.1

Among the respondents, 56.9% were female, and 43.1% were male, and the participants ranged in age from teenagers to individuals over 60 years old. Regarding education level, 80.9% of the respondents had a bachelor's degree. The proportion of participants who had been using the OHC for <3 months, 3–6 months, and 6 months to 1 year was approximately one-quarter each; 14.4% had been part of the community for 1–2 years, and 10.5% had participated in the community longer than 2 years. Regarding the frequency of OHC usage, 26.5% of the respondents had used OHCs a few times a month, and 24.9% of the respondents had used OHCs once a month. A very small percentage of respondents used it less than once a month (7.5%) or several times a day (9.1%).

### Measurement Assessment

SmartPLS 3.0 was used to evaluate the measurement validity and reliability. Compared with other data analysis software, SmartPLS has some advantages. First, PLS estimates the measurement and structural models simultaneously (Gefen et al., 2000). Second, PLS avoids inadmissible solutions and factor indeterminacy. It is suitable for testing complicated relationships (Sun et al., 2016).

We analyzed the reliability and validity of the constructs. The reliability of a construct refers to the degree of internal consistency among the instrumental items (Straub et al., 2004). Reliability can be tested by combining two indicators: composite reliability (CR) and Cronbach's α. A high value of these two indicators entails high internal consistency between the variables of the items, thus indicating higher reliability. In general, the CR value and Cronbach's α should be >0.7 (Straub et al., 2004). As shown in Table 3, the Cronbach's α of all constructs are between 0.802 and 0.906, which are greater than the recommended value of 0.7. The CR values are between 0.870 and 0.934, which are also greater than the benchmark value of 0.7. Therefore, the constructs involved in this study had good credibility.

**Table 3:** Test results of internal reliability and convergent validity.

**Construct**	**Items**	**Cronbach's Alpha**	**Convergent Validity**		
			**Factor Loading**	**CR**	**AVE**
TPC	TPC1	0.906	0.858	0.934	0.781
	TPC2		0.890		
	TPC3		0.873		
	TPC4		0.912		
RPC	RPC1	0.826	0.843	0.884	0.656
	RPC2		0.777		
	RPC3		0.828		
	RPC4		0.790		
CI	CI1	0.802	0.759	0.870	0.626
	CI2		0.809		
	CI3		0.772		
	CI4		0.823		
KSS	KSS1	0.829	0.879	0.897	0.745
	KSS2		0.843		
	KSS3		0.866		
KC	KC1	0.834	0.834	0.889	0.667
	KC2		0.805		
	KC3		0.800		
	KC4		0.828		

*TPC, Transactional Psychological Contracts; RPC, Relational Psychological Contracts; CI, Community Identification; KSS, Knowledge Sharing Self-efficacy; KC, Knowledge Contribution*.

The validity of the construct refers to the degree to which the items can accurately measure the construct that needs to be measured (Straub et al., 2004). Validity includes convergent validity and discriminant validity. Convergent validity can be tested by two indicators: average variance extracted (AVE) and factor loading. The AVE must be >0.5, and the factor loading must be greater than the benchmark value of 0.7 (Straub et al., 2004). As shown in the table, the AVE values of all constructs are between 0.626 and 0.781, which are greater than the benchmark value of 0.5. The factor loading values of all constructs are between 0.759 and 0.912, which are greater than the benchmark value of 0.7. In summary, all the constructs in this study have good convergent validity.

Discriminant validity refers to the degree of difference between multiple variables. It can be judged by the following two criteria. First, the square root of the AVE of each construct is compared with the correlation coefficient of other constructs. Second, the loading of each construct on its corresponding factor is compared with the loading of the cross factor on other factors. As shown in Table 4, the square roots of the AVE of all constructs are greater than the correlation coefficient between each construct and other constructs, indicating that the research model has good discriminant validity (Fornell and Larcker, 1981). Moreover, the loadings of all constructs on their corresponding factors exceeded the cross-loading on other factors (shown in Table 5), thus confirming the discriminant validity of the research model (Hair et al., 2003).

**Table 4:** Correlations among constructs.

**Construct**	**Mean**	**S.D**.	**TPC**	**RPC**	**CI**	**KSS**	**KC**
TPC	3.64	0.90	**0.884**				
RPC	5.45	1.02	−0.398	**0.810**			
CI	3.95	0.69	−0.451	0.622	**0.791**		
KSS	5.24	1.14	−0.313	0.394	0.399	**0.863**	
KC	5.50	1.01	−0.529	0.515	0.547	0.612	**0.817**

*Diagonal elements are square roots of AVEs. TPC, Transactional Psychological Contracts; RPC, Relational Psychological Contracts; CI, Community Identification; KSS, Knowledge Sharing Self-efficacy; KC, Knowledge Contribution*.

**Table 5:** Item cross-loadings.

**Items**	**TPC**	**RPC**	**CI**	**KSS**	**KC**
TPC1	**0.858**	−0.385	−0.423	−0.295	−0.526
TPC2	**0.890**	−0.354	−0.395	−0.227	−0.402
TPC3	**0.873**	−0.320	−0.385	−0.285	−0.427
TPC4	**0.912**	−0.342	−0.388	−0.293	−0.500
RPC1	−0.315	**0.843**	0.561	0.391	0.492
RPC2	−0.316	**0.777**	0.477	0.301	0.394
RPC3	−0.329	**0.828**	0.496	0.293	0.380
RPC4	−0.333	**0.790**	0.475	0.280	0.392
CI1	−0.283	0.454	**0.759**	0.260	0.324
CI2	−0.351	0.463	**0.809**	0.331	0.441
CI3	−0.315	0.522	**0.772**	0.286	0.386
CI4	−0.453	0.525	**0.823**	0.370	0.546
KSS1	−0.314	0.327	0.348	**0.879**	0.505
KSS2	−0.253	0.360	0.369	**0.843**	0.517
KSS3	−0.247	0.333	0.319	**0.866**	0.558
KC1	−0.447	0.454	0.472	0.504	**0.834**
KC2	−0.420	0.412	0.423	0.492	**0.805**
KC3	−0.428	0.383	0.413	0.491	**0.800**
KC4	−0.434	0.433	0.476	0.512	**0.828**

*TPC, Transactional Psychological Contracts; RPC, Relational Psychological Contracts; CI, Community Identification; KSS, Knowledge Sharing Self-efficacy; KC, Knowledge Contribution*.

Potential biases resulting from common-method variance (CMV) may exist since self-reported data were used in this study. To rule out CMV, Harman's single-factor test was used to analyze the variance proportion of a single factor. The basic assumption of Harman's single-factor test is that one general factor will account for the majority (50%) of the covariance among the measures (Podsakoff et al., 2003). The results show that the first extracted component, with the largest eigenvalue, explains 31.06% of all variance, suggesting that CMV is not an issue in this study.

## Structural Model Results

**Figure 2:**
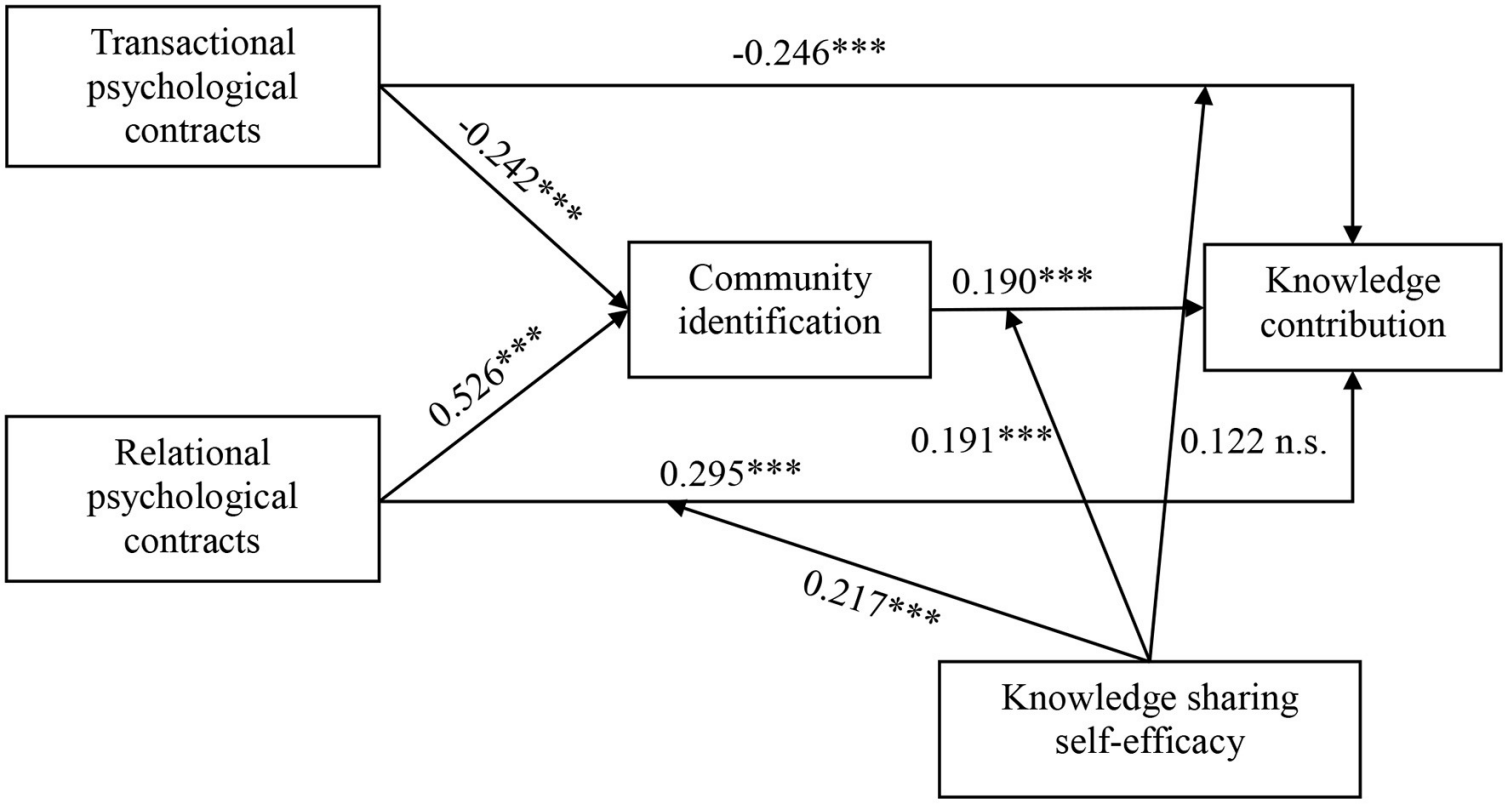
Path coefficient of structural model. **p < 0.01, ***p < 0.001.

A structural equation modeling analysis is conducted on SmartPLS 3.0 to test the relationships in the research model. SmartPLS does not provide information about fitness indices of the entire model but presents the R-square of each dependent variable. The R-square of CI is 0.437, indicating that the variation that can be explained is 43.7%. The R-square of KC is 0.677, indicating that the variation that can be explained is 67.7%. The result presents a good model fit. Figure 2 presents the path coefficients between each pair of constructs in the structural model. The figure also shows the results of the structural path analysis. The fitness of the overall model is fairly good.

As shown in Figure 2, the path coefficient between transactional psychological contracts and knowledge contribution (β = −0.246, *p* < 0.001) is negatively significant, thereby supporting Hypothesis 1. The path coefficient between relational psychological contracts and knowledge contribution is positively significant (β = 0.295, *p* < 0.001), which supports Hypothesis 2. In addition, the path coefficient between transactional psychological contracts and community identification (β = −0.242, *p* < 0.001) is negatively significant, whereas the path coefficient between relational psychological contracts and community identification (β = 0.526, *p* < 0.001) is positively significant, thereby validating Hypothesis 3 and Hypothesis 4. The significant positive path coefficient between community identification and knowledge contribution (β = 0.190, *p* < 0.001) supports Hypothesis 5.

Prior studies have suggested that psychological contracts can lead to employees' community identification, thereby motivating their knowledge contribution behaviors (Liu et al., 2020). Given this, our study examines how community identification mediates the relationship between psychological contracts and knowledge contribution by using a bootstrapping method to verify the mediation effect. Using the bootstrapping method in SmartPLS to run 5,000 times, the level value under 95% confidence is shown in the following table.

Table 6 reveals that transactional psychology contracts have a relatively significant indirect effect (β = −0.046, *p* < 0.001) on knowledge contribution via community identification, thereby supporting Hypothesis 6a. In addition, relational psychology contracts have a significant indirect effect (β = 0.100, *p* < 0.001) on knowledge contribution via community identification, thus supporting Hypothesis 6b.

**Table 6:** Mediating effects of organizational identification.

**Path**	**β**	**STDEV**	**T**	***P***	**Confidence Intervals**	
					**2.5%**	**97.5%**
RPC ->CI ->KC	0.100	0.025	3.978	0.000	0.057	0.157
TPC ->CI ->KC	−0.046	0.014	3.366	0.001	−0.077	−0.023

*TPC, Transactional Psychological Contracts; RPC, Relational Psychological Contracts; CI, Community Identification; KC, Knowledge Contribution*.

**Figure 3:**
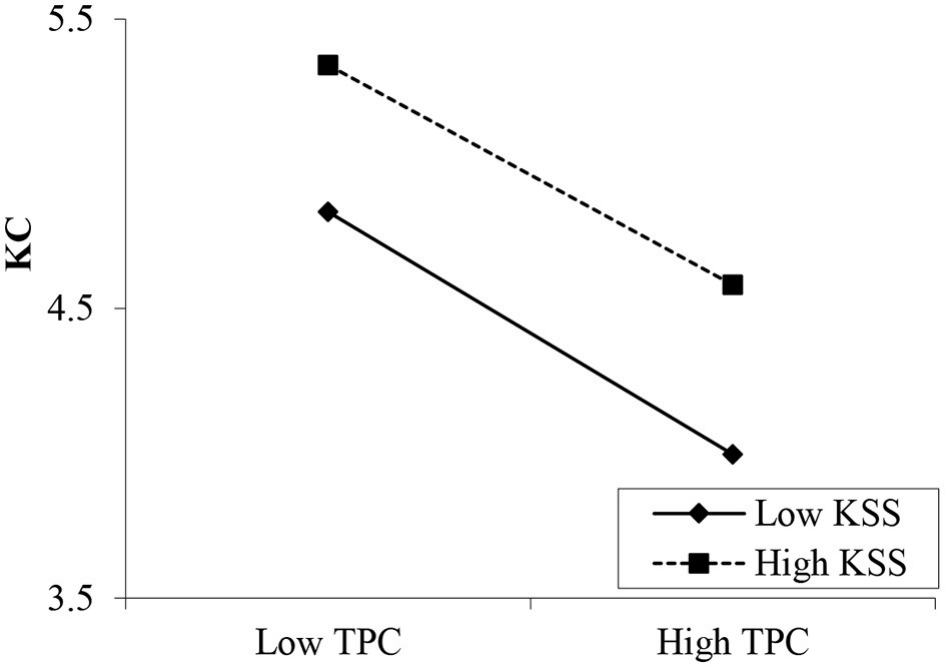
Moderating effect of knowledge sharing self-efficacy (KSS) between transactional psychological contracts (TPC) and knowledge contribution (KC).

**Figure 4:**
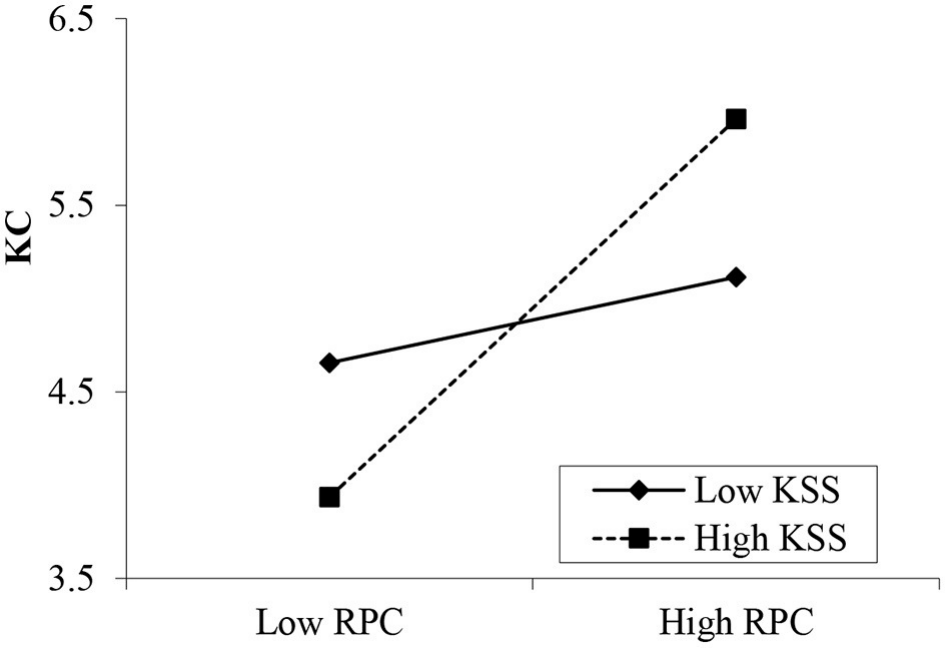
Moderating effect of knowledge sharing self-efficacy (KSS) between relational psychological contracts (RPC) and knowledge contribution (KC).

**Figure 5:**
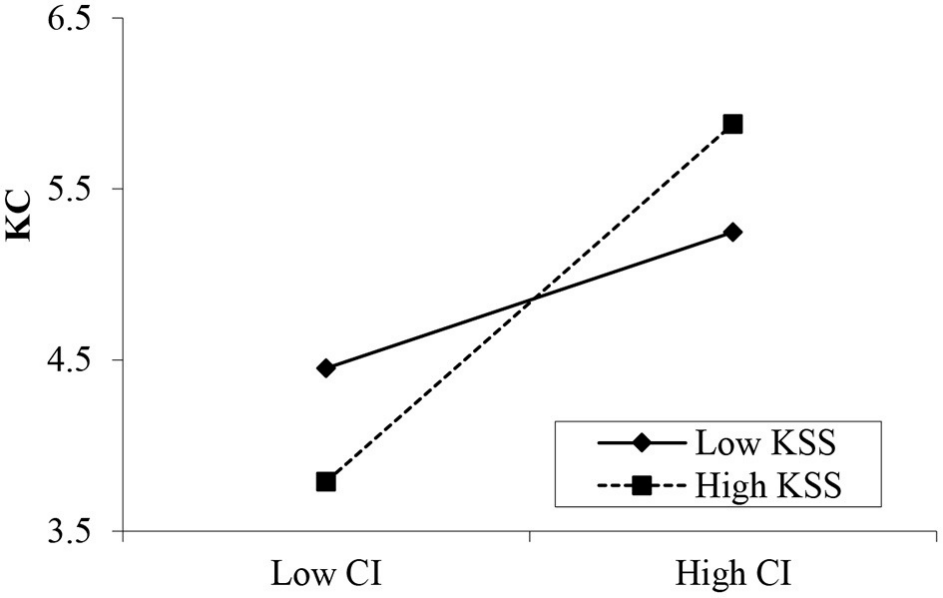
Moderating effect of knowledge sharing self-efficacy (KSS) between community identification (CI) and knowledge contribution (KC).

Hypotheses 7a and 7b propose the moderating effects of knowledge sharing self-efficacy on the relationships between psychological contracts and knowledge contribution. The results in Table 7 show that the interaction between transactional psychological contracts and self-efficacy is insignificant (β = 0.122, *p* > 0.05; Figure 3). However, the interaction between relational psychological contracts and knowledge sharing self-efficacy is significant (β = 0.217, *p* < 0.001; Figure 4). Thus, H7b is supported, but H7a is rejected. Moreover, the interaction between community identification and self-efficacy was significant (β = 0.191, *p* < 0.001, Figure 5). Therefore, Hypothesis 7c is supported.

**Table 7:** Moderating effects of knowledge sharing self-efficacy.

**Path**	**β**	**STDEV**	**T**	***P***
CI*KSS ->KC	0.191	0.051	3.774	0.000
RPC*KSS ->KC	0.217	0.063	3.444	0.001
TPC*KSS ->KC	0.122	0.080	1.518	0.129

*TPC, Transactional Psychological Contracts; RPC, Relational Psychological Contracts; CI, Community Identification; KSS, Knowledge Sharing Self-efficacy; KC, Knowledge Contribution*.

## Discussion

This study explores the influence of two types of psychological contracts between community members and OHCs on knowledge contribution. It also examines the mediating role of community identity and the moderating role of knowledge self-efficacy.

The structural equation modeling method is adopted to examine the theoretical hypotheses. The test results show that the two types of psychological contracts play different roles in online health community members' knowledge contribution. Specifically, transactional psychological contracts have a significantly negative impact on knowledge contribution, whereas relational psychological contracts have a positive impact on knowledge contribution. These results echo the findings of previous studies that focused on offline organizations (Turnley et al., 2003; Lu et al., 2016; Soares and Mosquera, 2019). By investigating frontline hotel employees, Lu et al. (2016) found that relational psychological contracts are more effective in motivating employees' high performance. In contrast, transactional psychological contracts are negatively related to employees' in-role and extra-role performance. Similar results were also obtained with samples from nonprofit organizations. Soares and Mosquera (2019) investigated members from military departments and found that members who experience a relational psychological contract have high work engagement, while those who experience a transactional psychological contract have low work engagement. These findings also confirmed the argument of Turnley et al. (2003), who proposed and clarified that psychological contract fulfillment regarding relationships is positively related to employees' performance. In short, the findings of this study demonstrate that both the negative impact of transactional contracts and the positive impact of relational contracts on individuals' performance also exist in online health communities. Compared to transactional psychological contracts, relational psychological contracts play a positive role in members' knowledge contribution.

Furthermore, the two dimensions of psychological contracts affect knowledge contribution through community identification. Community members who originally only pay attention to or are mainly concerned about the exchange of benefits rarely develop a sense of belonging and loyalty to the community, which is not conducive to inspiring the willingness to contribute their knowledge. However, individuals' relational psychology contract contributes to the formation of their community identification and thereby plays a positive role in their knowledge contribution. This result is similar to those of previous studies (Bergami and Bagozzi, 2000; van den Hooff et al., 2003; Chiu et al., 2015; Lu et al., 2016). For example, Lu et al. (2016) proposed and empirically examined that organizational identification is a critical psychological factor that translates psychological contracts into individuals' behavior. Nevertheless, this study is distinct from prior studies. Unlike the construct of transactional psychological contract that emphasizes the unwritten agreement of exchanges based on monetary or materialistic drivers in prior studies, the transactional psychological contract in this study focuses on the exchange of information, knowledge, and help. The findings of this study indicate that knowledge acquisition motivation and obligation to provide help or knowledge to others neither help cultivate OHC users' community identification nor lead to high intention to contribute knowledge. Similarly, the relational psychological contract is measured with items that are unlike those used in studies that investigate offline organizations. Traditional relational psychological contracts are formed and developed on the basis of real-world interactions, such as interactions among organization members or between individuals and an organization. By contrast, the relational psychological contract in this study emphasizes the virtual relationships in online communities, which are positively related to individuals' community identification. Hence, this study broadens the application context of psychological contracts by introducing it from offline organizations to online organizations. In conclusion, although the measurements of psychological contract vary, the influence mechanism of psychological contracts on members' attitude and behavior is the same.

In addition, we found that knowledge sharing self-efficacy has a positive moderating effect on the path of relational psychological contracts to knowledge contribution. It also has a positive effect on the influence of community identification on knowledge contribution. However, no significant moderating effect exists on the influence path of the transactional psychological contract on the contribution of knowledge. That is to say, knowledge-sharing self-efficacy has a positive interactional effect with affective factors, such as relational psychological contract and community identification, on individuals' OCBs. However, it does not change the negative impact of transactional psychological contracts.

### Theoretical Significance of Research Results

This study generates several important theoretical contributions. First, it enriches research about psychological contracts and knowledge contributions in OHCs. In the past, most studies situate the exploration of the relationship between psychological contracts and knowledge contribution in the context of companies (Riikka and Läms, 2014). However, with the popularization of the Internet in recent years, an increasing number of people have begun to conduct activities in virtual communities, such as SNSs, online brand communities, and online Q&A communities, which also include OHCs. In OHCs, people who have had similar experiences have a tendency to share their experiences and knowledge. Therefore, exploring the influencing factors of knowledge contribution in OHCs has important theoretical significance. This study verifies the impact of the psychological contract of the transactional dimension and relational dimension on the knowledge contribution activities of OHCs through empirical analysis, and community identification plays an intermediary role. First, transactional psychological contracts negatively affect OHC users' knowledge contribution, whereas relational psychological contracts positively affect their knowledge contribution. Second, transactional contracts are negatively related to users' community identification, whereas relational contracts lead to higher community identification, which thereby enhances users' intention to share and contribute knowledge. Previous studies have confirmed these relationships in enterprises but rarely in the context of OHCs. This study provides a new perspective to investigate OHC users' community participation.

Second, we include knowledge sharing self-efficacy into the research framework to explore its moderating effect. In terms of knowledge contribution behavior, knowledge sharing self-efficacy plays a vital role in individuals' knowledge-sharing activities. It can diversify members' knowledge-contributing behaviors (Hsu et al., 2007). Health professionals and users who are relatively knowledgeable and familiar with using information technology have reasonable confidence to provide accurate and useful medical information to others, and other patients can make health decisions on the basis of the information they obtain from the OHCs (Zhang et al., 2017). Users' high self-efficacy promotes their knowledge contribution behavior and makes them perceive achievement from their knowledge sharing behavior, especially when their knowledge can help others. Specifically, when OHC users regard themselves as members or citizens of the OHC, their knowledge sharing self-efficacy positively interacts with their relational psychological contract and community identification to devote themselves to the community. To conclude, knowledge sharing self-efficacy can enhance the effects of relational contracts and community identification on knowledge contribution.

### Practical Significance of Research Results

A clear understanding of the psychological mechanisms through which knowledge contribution operates is critical to the promotion of willingness to offer contribution. Previous research suggests that by contributing a part of their unique knowledge, knowledge contributors give up sole claims to the benefits stemming from such knowledge. Methods to promote knowledge contribution must be sought. This study provides some practical ways.

First, attention must be given to the construction of relational psychological contracts. In this study, we found that relational psychological contracts have a positive effect on knowledge contribution. Thus, the managers of OHCs should establish a good communication mechanism to promote users' communication among community members. China is a society that places great emphasis on harmonious coexistence. Within such a cultural background, the key to integrating into the community, and being willing to contribute knowledge is to establish a good relationship with other members of the community. Generally, people hope to establish a friendly relationship with others and receive care, friendship, support, cooperation, and appreciation from others. In particular, in the specific virtual community of OHCs, users can meet numerous people who share similar experiences. In the process of mutual communication, empathy enables them to understand one another and produce feelings of mutual respect. In such an environment, community members learn to trust others and their community. They are also more willing to contribute their own knowledge. For example, in addition to answering and commenting on the public questions of other members in the OHCs, community members can also send private messages, likes, and give virtual gifts. They can also create different groups and circles to build a social network model to promote communication among community members.

The second method is to build a good corporate culture and values. The core of corporate culture is to share common values, which guide members' behavior. In an OHC, an atmosphere that encourages knowledge contribution can encourage community members to share and exchange their knowledge and experience more naturally. One of the purposes of community members using the OHC is to obtain insight from the experiences of people with similar cases or the knowledge provided by professionals. By combining personal goals and community goals through the construction of community culture and values, community members subtly accept and agree with the values of the community and develop a sense of belonging, loyalty, and emotional identification. In the process of communicating with others, they can take pride in contributing knowledge and creating a sense of accomplishment, which encourages community members to be willing to exchange and share knowledge continuously. Contributing their talents to common interests is also conducive to a good community atmosphere and achieving win-win cooperation.

Last, the managers of OHCs should raise their perceptions of knowledge self-efficacy. Our findings indicate that the knowledge contribution of self-efficacy plays an important role in promoting individuals' willingness to offer knowledge contribution as the expression of their identification with the community. For instance, organizations such as Amazon.com regularly recognize their top reviewers, serving as a way to enhance the self-efficacy of these knowledge contributors (Kankanhalli et al., 2005). Thus, they can deliver a message to community members who share their knowledge to make them aware that their knowledge contribution means much to OHCs.

## Conclusion

This study investigates OHC users' knowledge contribution behavior from the perspective of psychological contracts, which has been widely discussed in other organizations but not in OHCs. Scales of transactional psychological contract, relational psychological contract, community identification, knowledge contribution, and self-efficacy are adopted from prior studies and adjusted to this study. A total of 362 valid samples from OHCs, such as Dingxiangyuan and Manyoubang, from China are used in the analysis. The reliabilities of the constructs are confirmed according to the values of composite reliability (CR) and Cronbach's α. Moreover, convergent validity is confirmed based on the factor loadings and AVEs. Meanwhile, by comparing the square root of AVE of each construct with the corresponding correlations, as well as observing the item cross-loading, the discriminant validity among constructs is confirmed. The results of the structural model test confirm our propositions on the relationships among OHC users' psychological contracts, community identification, and knowledge contribution (H1 to H6). Moreover, the moderating effects of self-efficacy in H7b and H7c are also verified. The results of this study have theoretical and practical implications. Despite the contribution of this study, some limitations exist because of the research design and process. First, this study does not analyze users' different types of psychological contracts and their community identification under various lengths of membership. The length of an individual's membership in an organization, to some extent, determines their psychological contacts with the organization (Conway and Coyle-Shapiro, 2012). In addition, long-tenured members are more committed to an organizational mission and thereby more willing to contribute to the organization (Lee et al., 2018). Thus, the role of the length of membership in the OHCs should be considered in future work. Second, OHCs are special communities in which most users are patients suffering from certain diseases. Whether they achieve health improvements after joining OHCs may also moderate the relationship between their psychological contract and knowledge contribution. Third, the responses of the participants of this study were collected from several OHCs. Rewarding or motivating systems may differ from one another and should be considered as a control variable. All the above shortcomings of this study are expected to be further investigated in future works.

## Data Availability Statement

The raw data supporting the conclusions of this article will be made available by the authors, without undue reservation.

## Ethics Statement

The studies involving human participants were reviewed and approved by the Ethics Review Board of the College of Economics and Management of Nanjing University of Aeronautics and Astronautics. Written informed consent for participation was not required for this study in accordance with the national legislation and the institutional requirements.

## Author Contributions

WL, XC, XL, and XF designed the study. XC and XL collected and analyzed the data, and drafted the manuscript. WL and XF contributed to manuscript revision and read and approved the submitted version. All authors contributed to the article and approved the submitted version.

## Conflict of Interest

The authors declare that the research was conducted in the absence of any commercial or financial relationships that could be construed as a potential conflict of interest.
