# Corrigendum: Exploring the Relationship Between Users' Psychological Contracts and Their Knowledge Contribution in Online Health Communities

**DOI:** 10.3389/fpsyg.2021.716702

**Published:** 2021-06-30

**Authors:** Wenlong Liu, Xinting Chen, Xuanyu Lu, Xiucheng Fan

**Affiliations:** ^1^College of Economics and Management, Nanjing University of Aeronautics and Astronautics, Nanjing, China; ^2^School of Management, Fudan University, Shanghai, China

**Keywords:** online health community, transactional psychological contracts, relational psychological contracts, community identification, knowledge sharing self-efficacy, knowledge contribution

In the original article, there was an error. It should be “A total of 362 valid responses…” in the fourth line of the Abstract, not “367 valid responses…”. We rechecked the “Survey Administration” part of the manuscript as well as our data and confirmed that it is 362 valid responses.

A correction has been made to *Abstract*. The corrected section is shown below.

The knowledge contribution of members is essential and beneficial to both the business and users of online health communities (OHCs). This study explores and tests the effects of OHC users' psychological contracts on their community identification and knowledge-sharing behavior. A total of 362 valid responses from several well-known OHCs in China are used in the data analysis. The results of the path analysis with structural equation modeling show that users' transactional psychological contracts have a negative effect on their knowledge contribution both directly and indirectly by weakening their community identification. In contrast, users' relational psychological contracts can lead to increased active knowledge contributions both directly and indirectly by enhancing their community identification. Knowledge sharing self-efficacy can strengthen the relationship between relational psychological contracts and knowledge contributions, and the relationship between community identification and knowledge contributions. However, it has no significant impact on the path from transactional psychological contracts to knowledge contribution. The implications and direction of future works are presented on the basis of the results of the empirical analysis.

The authors apologize for this error and state that this does not change the scientific conclusions of the article in any way. The original article has been updated.

